# Quincke-edema induced by chlorpromazine: About two cases

**DOI:** 10.1192/j.eurpsy.2021.1288

**Published:** 2021-08-13

**Authors:** S. Brahim, W. Bouali, M. Henia, A. Abid, L. Zarrouk

**Affiliations:** 1 Psychiatry, University Hospital of Mahdia, Tunisia., chebba, Tunisia; 2 Department Of Psychiatry, University Hospital Of Mahdia, Tunisia., Psychiatry, Mahdia, Tunisia; 3 Anesthesia, University Hospital of Mahdia, Tunisia., mahdia, Tunisia

**Keywords:** chlorpromazine, clinical case, Pharmacology, Quincke-edema

## Abstract

**Introduction:**

Quincke-edema has been specifically associated with using certain drugs including chlorpromazine as detailed through two clinical cases.

**Objectives:**

Illustration of two clinical cases about angioedema induced by Chlorpromazine.

**Methods:**

We reviewed clinical data from two patients who committed a suicide attempt and then transferred to the psychiatry department after their somatic stabilization: the first was 27-year-old followed in psychiatry since childhood for intellectual deficiency and admitted to the emergency department for the suicide attempt by taking 14 tablets of chlorpromazine 100 mg and the second was a 20-year-old patient, admitted to the emergency department for suicide attempt by Raticid.

**Results:**

The first patient presented a delusional persecution-themed syndrome with auditory hallucinations. Therefore, he was initially put on injectable treatment with Haloperidol 15mg and Diazepam 30mg then oral relay after 48h by Risperidone 4 mg and Chlorpromazine 200 mg. On the fourthday of his hospitalization, he presented a Quincke edema without laryngeal impairment. We stopped chlorpromazine and eliminated the other causes of this edema, resulting in a gradual regression of symptomatology. The second patient was put on chlorpromazine. On the second day, the patient presented a Quincke edema without laryngeal impairment. Somatic examination and biological exploration did not reveal any abnormalities. We stopped chlorpromazine and put the patient on Dexamethasone 3 days in a row resulting in a good outcome.

**Conclusions:**

These two cases identified a Quincke-edema reaction associated with the use of Chlorpromazine, this complication can lead to life-threatening manifestations and warrants greater awareness of the potential for recurrence.

